# Interrater Reliability of Diagnostic Methods in Traditional Indian Ayurvedic Medicine

**DOI:** 10.1155/2013/658275

**Published:** 2013-09-26

**Authors:** Vrinda Kurande, Anders Ellern Bilgrau, Rasmus Waagepetersen, Egon Toft, Ramjee Prasad

**Affiliations:** ^1^Department of Health Science & Technology, Faculty of Medicine, Aalborg University, Frederik Bajers Vej 7D, 9220 Aalborg East, Denmark; ^2^Department of Mathematical Sciences, Aalborg University, Frederik Bajers Vej 7G, 9220 Aalborg East, Denmark; ^3^Department of Haematology and Aalborg Hospital Science and Innovation Center (AHSIC), Aalborg University Hospital, Sdr. Skovvej 15, 9000 Aalborg, Denmark; ^4^Center for TeleInFrastruktur (CTIF), Aalborg University, Niels Jernes Vej 12, 9220 Aalborg East, Denmark

## Abstract

This study assesses the interrater reliability of Ayurvedic pulse (*nadi*), tongue (*jivha*), and body constitution (*prakriti*) assessments. Fifteen registered Ayurvedic doctors with 3–15 years of experience independently examined twenty healthy subjects. Subjects completed self-assessment questionnaires and software analyses for *prakriti* assessment. Weighted kappa statistics for all 105 pairs of doctors were computed for the pulse, tongue, and *prakriti* data sets. According to the Landis-Koch scale, the pairwise kappas ranged from poor to slight, slight to fair, and fair to moderate for pulse, tongue, and *prakriti* assessments, respectively. The average pairwise kappa for pulse, tongue, and *prakriti* was 0.07, 0.17, and 0.28, respectively. For each data set and pair of doctors, the null hypothesis of random rating was rejected for just twelve pairs of doctors for *prakriti*, one pair of doctors for pulse examination, and no pairs of doctors for tongue assessment. Thus, the results demonstrate a low level of reliability for all types of assessment made by doctors. There was significant evidence against random rating by software and questionnaire use and by the diagnosis preferred by the majority of doctors. *Prakriti* assessment appears reliable when questionnaire and software assessment are used, while other diagnostic methods have room for improvement.

## 1. Introduction

In Ayurveda, the physician's bimodal approach of clinical examination (disease diagnosis and patient diagnosis) is used to determine the root cause of the disease and to determine the treatment selection [1]. Diagnostic decision making in Ayurveda is a complex process. It includes interpretation through an intrinsic understanding of many factors involved in disease manifestation such as “body humors” (*dosha*), body tissues (*dhatu-s*), excretory products (*mala-s*), digestive power (*agni*), and body channels (*srota-s*). Moreover, Ayurveda also takes into account pathogenic factors, season, and a patient's entire course of action (diet, drug, and regimen compatible with the constitution) for the expression of the disease. An Ayurvedic clinical examination includes three diagnostic methods (*trividha pariksha*): inspection, interrogation, and palpation. Inspection involves observation of the body parts, for example, skin, hair, eyes, and tongue. Comprehensive understanding of medical history, symptoms, and psychological and physiological characteristics are covered during the interrogation. Palpation includes pulse, and palpation of body parts (abdominal palpation, skin, etc.). Based upon a conventional medical diagnosis, treatment and choice of herbs/compound formulae are prescribed. However, very little is known about the reliability of Ayurvedic diagnostic methods.

In the clinical settings, interrater reliability is the degree to which two or more raters agree on a diagnosis of the same subject under identical assessment conditions. Reliability studies are necessary because they provide information about the quality of measurements and also play an important role in the process of developing effective diagnostic procedures [2]. 

The Ayurvedic concepts of physiology, pathology, diagnosis, medicine, and therapeutics are based on the doctrine of the three *doshas* (Appendix A). Every *dosha* is believed to have inherent attributes, which are expressed in the physical, psychological, and physiological characteristics of an individual. The authentic Ayurvedic text *Charak samhita*, *Sushruta samhita* explicitly explains how to identify *dosha* properties through signs and symptoms leading to a manifestation of *prakriti *and diseases. Recently, few studies observed genetic bases for *prakriti* [3]. Construct of *prakriti* has been correlated to human leukocyte antigen (HLA) gene polymorphism [4]. Another study reported that biochemical profiles and hematological parameters exhibited differences between *prakriti* types [5]. A significant association between CYP2C19 genotype and major classes of *prakriti* types was observed in [6]. Another study showed platelet aggregatory response, and its inhibition by aspirin varied in the different *prakriti* subtypes [7]. This *prakriti*-related evidence is likely to have a significant impact on personalized medicine. However, there is a lack of quantitative studies such as reliability of *prakriti* assessment. Based on the combination of one or more bioentities, seven types of *prakriti *are described: *vataja, pittaja, kaphaja, vatapittaja, vatakaphaja, pittakaphaja,* and *vatapittakaphaja*. *Prakriti *analysis helps in prioritizing any nurturing, preventive, and curative regimen specific to an individual. Thus, *prakriti*-based prescription helps to enhance the therapeutic effect of a regimen and to reduce the unwanted effects of the drug. For more reliable diagnosis results, analysis of the *prakriti *assessment itself is essential [8–10]. *Prakriti *represents a natural combination of one or more *doshas*. In addition, the current status or level of the *dosha* can be diagnosed by pulse examination (*nadi pariksha*) [11]. The tongue diagnosis (*jivha pariksha*) is also a useful method. Tongue examination helps in assessing “status of digestion” [12]. Visual inspection of the tongue includes observation of tongue color, shape, and tongue coating. According to Ayurveda, a malfunctioning of digestive/biological fire (*agnimandya*) lies at the root of all diseases. The decreased functioning of the biological fire (*mandagni*) causes the improper digestion of the food and leads to the formation of an autotoxin (*ama*) [13]. This autotoxin is mixed with the bioentities (*dosha*) and affects body tissues, thus vitiating/altering their qualities and leading to all kinds of pathological processes. Inspection of the tongue coating in the early stages is useful to diagnose an impairment of digestive fire, and intervention may prevent the further development of an autotoxin. Thus, changes in tongue coating with other symptoms of *ama* can provide significant information for different Ayurvedic diagnoses in the clinical practice.

Thus, pulse, tongue, and *prakriti *assessment are integral parts of an Ayurvedic diagnosis. To incorporate Ayurvedic diagnostic criteria into a clinical study to improve the confidence in the clinical findings, it is, however, necessary to confirm the validity and reliability of Ayurvedic diagnostic criteria [14, 15]. 

In the present study, we assess the interrater reliability of the pulse, tongue, and *prakriti *assessment through basic qualities of *vata*, *pitta,* and *kapha* and their combinations.

## 2. Materials and Methods

### 2.1. Pulse Examination Method

Pulse examination was done by placing the index, middle, and ring fingers at the root of the thumb of the subjects. For female subjects, the pulse was taken from the left side, and in the case of male subjects, the pulse was taken from the right side. The sensation of the *vata* pulse patterns is said to be like a snake's curved crawling; the sensation of the *pitta* pulse is described as a frog's jumping; and the sensation of the *kapha* pulse is described as a pigeon's or swan's smooth, slow movement. Each is felt by using the index, middle, and ring fingers, respectively. Detailed information of Ayurvedic pulse examination is given in [16].

### 2.2. Tongue Examination Method

Doctors assessed the degree of tongue coating. Tongue coating is defined as no coating (*niram jivha*), thin coating (*alpa sama*), and thick, sticky coating (*sama jivha*). 

### 2.3. *Prakriti* Examination Method

The *prakriti* has specific physical, physiological, and psychological characteristics based on *dosha* attributes. Detailed information is available in [9, 10, 17]. In this study, doctors assessed these characteristics by inspection, interrogation, and palpation to determine the *prakriti* for the subject (Table 1). After the clinical examination, doctors wrote their final *prakriti* assessment on the assessment form. 

### 2.4. ABC Questionnaire

The *prakriti* assessment questionnaire is a questionnaire for self-assessment. As no standard questionnaire is available for *prakriti*, we developed a new questionnaire in a simple, everyday language. The questionnaire consisted of a total of seventy-five items, comprising twenty-five items relating to each of the three *dosha* types—*vata*, *pitta,* and *kapha* types. Each item was composed of a three-level scale, which requires the subject to choose one of three possible answers: “not so much—1," “normally medium—2," and “yes, very much—3" (Appendix B).

### 2.5. *Prakriti* Assessment Software

We used *Prakriti Vichaya*—*dosha prakriti*—(Constitution Assessment) software developed by Center for Development of Advanced Computing (CDAC, Pune). This is an extensive questionnaire based on age and gender groups. It gives a quantitative analysis based on anatomical, physiological, and psychological parameters. More information is available on their webpage [18]. 

### 2.6. Study Subjects

We included twenty healthy subjects (males: *n* = 10, females: *n* = 10, age range eighteen to twenty years) in the study. Subjects were randomly selected from second-year Ayurveda college students. Detailed information about the study design was given to all subjects prior to the study. To avoid a bias for *prakriti* assessment, the objectives of the study were not discussed with the students. Written consent was obtained from all subjects. An inclusion criterion was being eighteen years or older. All were in good health, and none was on medication.

### 2.7. Ayurvedic Doctors

**Figure 1:**
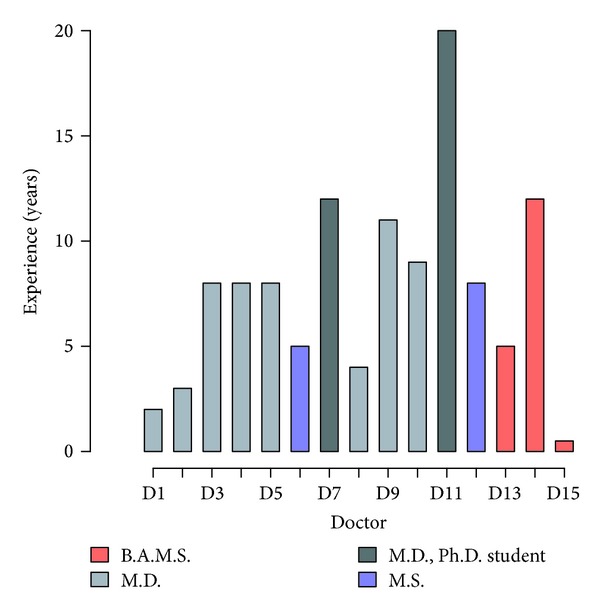
The experience and educational level of the 15 doctors.

Fifteen registered doctors, who have been practicing in Sri Sri College of Ayurvedic Science & Research Hospital, conducted the study. Ten were M.D. (Ayurveda) holders, two had M.S. in Ayurveda, and three had a B.A.M.S. (Bachelor of Ayurveda, Medicine and Surgery) in Ayurveda and had completed a pulse diagnosis course (Figure 1).

### 2.8. Study Procedure

**Figure 2:**
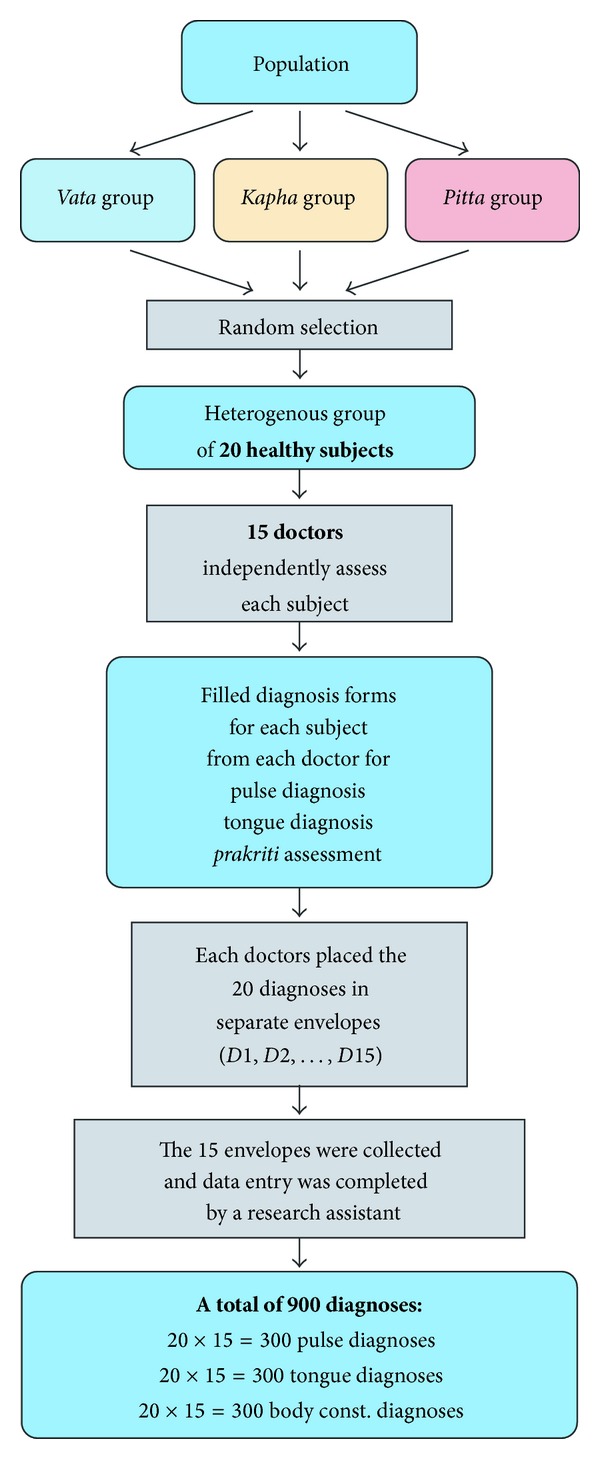
Flow chart of study procedure.

The study was conducted at Sri Sri College of Ayurvedic Science & Research Hospital in the morning. All subjects had been fasting for two hours. The doctors examined each subject independently. All doctors wrote their assessment of pulse, tongue, and *prakriti* on a separate assessment form for each subject. The flow chart of the study procedure is given in Figure 2. The study subjects completed self-assessments by completing a *prakriti* questionnaire and a software questionnaire within one week of the examination.

### 2.9. Statistical Analysis

**Figure 3:**
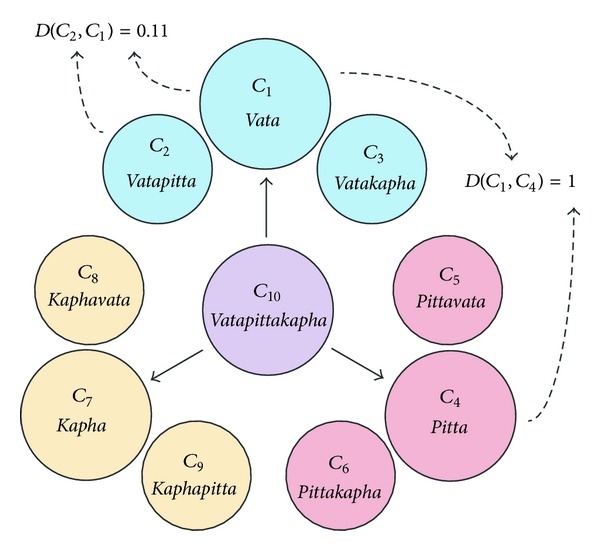
Distance between two classes, *D*: distance, *C*: classes.

Both pulse patterns and the *prakriti* assessment are nominal variables corresponding to ten different classes. For the statistical analysis, we constructed a weighting of the ten classes based on their Ayurvedic interpretation. Weights were defined corresponding to mixtures of each of the basic types, *vata, pitta, *and *kapha,* to each class (Table 2). Based on the weightings, we used the distance measure defined in [11] between the two classes. The distance measure is between zero and one. The minimal distance of zero occurs when two diagnoses are identical, and the maximal distance of one occurs for two diagnoses, which have none of the basic types of three *dosha* in common. For instance, *D*(*vata*,* vata*) = 0, *D*(*vata*, *vatapitta*) = 0.11, and *D*(*vata*, *pittavata*) = 0.55, where *D* is the distance between two classes (Figure 3). All distances between the classes are given in Table 3 [11]. For tongue diagnosis, only three diagnosis classes are present. The chosen distances between these diagnostic classes are shown in Table 4. Cohen's weighted kappa statistic was used to measure interrater reliability [19]. Since the weighted kappa is only defined for two raters, all 105 possible pairwise comparisons were carried out for *prakriti*, tongue, and pulse diagnoses. The magnitudes of the weighted kappas were qualified by the Landis and Koch scale (LK scale) (Table 5) [20].

**Figure 4:**
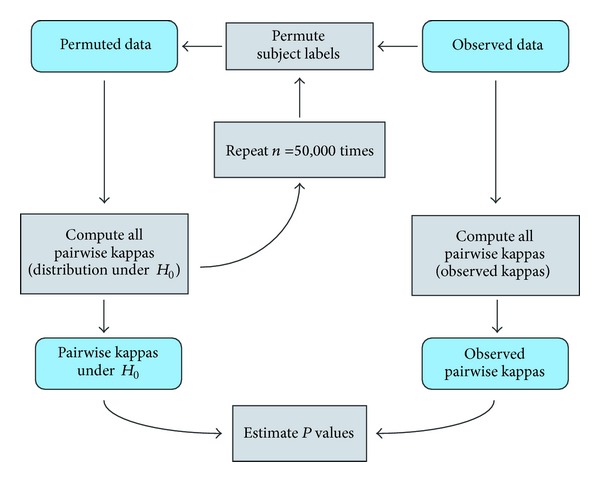
Flowchart for the permutation-type test for computing *P* values of pairwise kappas.

For each data set and each pair of doctors, we tested the null hypothesis of random rating, where the probability that the doctor assigns a particular diagnosis to a subject does not depend on the subject. A minimal requirement for agreement between doctors is that each of them performs significantly better than a random rating. Therefore, if the data do not show strong evidence against *H*_0_, this suggests a poor level of reliability. The *P* value can be viewed as an alternative to the Landis-Koch scale for interpreting the kappa statistics, where large *P* values correspond to low reliability. The *P* value for each pairwise kappa, that is, the probability of getting at least as favorable a weighted kappa as the observed, assuming *H*_0_, was computed by calculating the empirical distribution of the pairwise kappa under random permutation of subject for each doctor (Figure 4). Specifically, we used the estimate (*b* + 1)/(*n* + 1), where *b* is the number of pairwise kappas computed under permutation, that is, larger or equal to the observed, and *n* is the number of permutations. The number of permutations *n* used was 50,000. A Bonferroni correction was used to account for multiple hypothesis testing.

To get an overall level of reproducibility for pulse, tongue, and *prakriti *examinations, we computed the average of the 105 pairwise kappas for each diagnostic method. We also tested the hypothesis of random rating using the average kappas. Again, a permutation test was used as above; the permutations of ratings were within each doctor.

## 3. Results 

In this study, each doctor diagnosed *prakriti*, tongue, and pulse for twenty different subjects leading to a total of 300 (15 × 20) pulse diagnoses, 300 tongue diagnoses, and 300 *prakriti* diagnoses (Figure 2). 

### 3.1. Interrater Reliability of Pulse Examination

The percentages of pairwise kappas within each LK categories “poor,” “slight,” “fair,” and “moderate” were 40, 37, 20, and 3 percent, respectively (Table 5). None of the pairwise kappas were categorized as substantial or almost perfect/perfect. Forty percent of pairs had a negative value suggesting direct disagreement between doctors. Only one pair of doctors performed significantly better than random rating (Table 5).

The frequencies of diagnosis classes for all doctors for the pulse examination are shown in Figure 6(a). It shows that all classes except ten were used, and classes two, five, and six were reported the most frequently, while classes one, four, and nine were reported the least frequently. The average pairwise kappa (Table 6) for pulse examination was 0.07. Based on the average pairwise kappa, the hypothesis of random rating is rejected on the 5% level with a *P* value less than 2 × 10^−5^.

### 3.2. Interrater Reliability of Tongue Diagnosis

For tongue diagnosis, the percentages of kappas in the LK categories “poor,” “slight,” “fair,” and “moderate” were 16, 35, 41, and 6 percent, respectively (Table 5). None of the pairwise kappas were categorized as substantial or almost perfect/perfect. No significant evidence against the null hypothesis was found based on the separate pairwise kappas. All three tongue diagnostic classes were reported with class 2 (medium coating) as the most frequent (Figure 6(b)). The average kappa was 0.17, and based on this statistic, random rating is rejected with a *P* value less than 2 × 10^−5^ (Table 6).

### 3.3. Interrater Reliability of *Prakriti* Assessment

The level of reliability according to the LK scale is shown in Table 5. The percentages of kappas in the LK categories “poor,” “slight,” “fair,” “moderate,” and “substantial” were 9, 22, 44, 22, and 3 percent, respectively, for *prakriti* assessment. None of the pairwise kappas were categorized as almost perfect/perfect. The hypothesis of random rating was rejected for twelve pairs of doctors. The average kappa was 0.28 with a corresponding *P* value less than 2 × 10^−5^ (Table 6).

For each subject, we compared software and questionnaire diagnoses with the preferred assessment of the majority of the doctors. There was significant evidence against the hypothesis of random rating between software, questionnaire, and the preferred assessment of the majority of doctors. A moderate level of interrater reliability was present between the most frequent doctor's assessment and the software assessment, and likewise, a moderate level of reliability was found between the doctor's most frequent assessment and the questionnaire assessment. A fair level of reliability was found between the questionnaires and the software (Table 7). The diagnoses frequencies accumulated by the doctors for *prakriti* assessment show that all classes except combination of three *doshas *(*tridoshaja*) were used and that *vatapittaja*, *pittavataja*, *pittakaphaja*, and *kaphapittaja* were used most frequently while *kaphavattaja, vatakaphaja*, and *pittaja* were used least frequently (Figure 6(c)).

**Figure 5:**
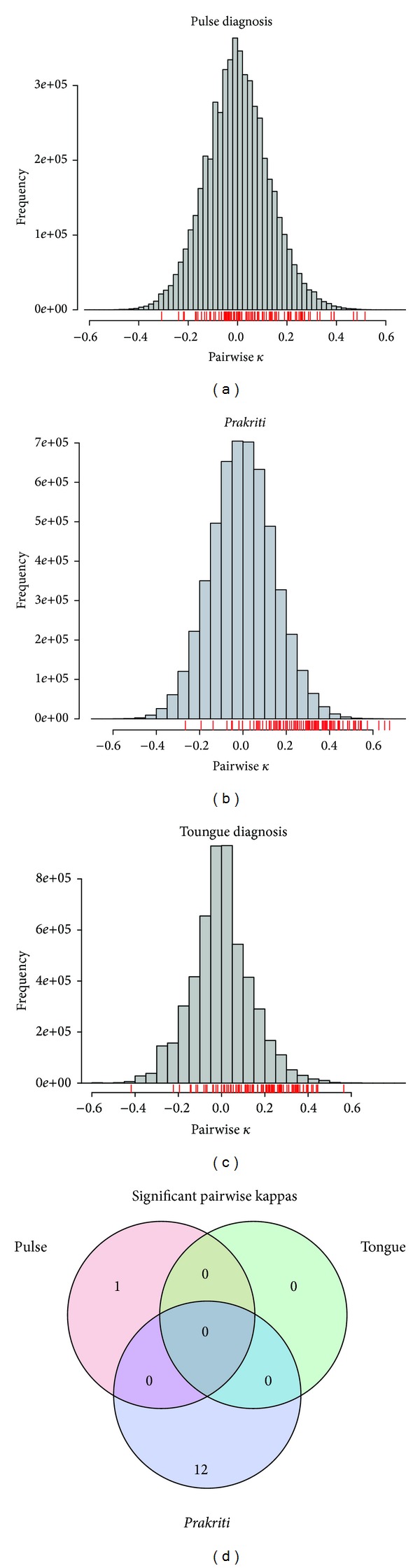
(a) to (c) show the histogram of all the pairwise kappas under permutation for the three datasets. The red “rug” (or ticks) below each plot shows the observed 105 pairwise kappas for comparison. (d) shows a Venn diagram of the significant *P* values in each dataset.

The distribution of all pairwise kappas for pulse, tongue, and *prakriti *assessment is seen in Figure 5.  Figure 5(d) shows a Venn diagram of the significant *P* values in each dataset. No pairwise kappa was significant in more than one dataset. There is no common significant *P* value for any diagnosis. For example, the pair of doctors who did better for *prakriti* assessment (12 significant *P* values) did not show the same result for tongue or pulse examination.

To see whether pairs of doctors with a high degree of reliability (i.e., a high pairwise kappa) in one dataset also concur in another dataset, scatter plots of the pairwise kappa values between different diagnoses were made and shown in Figure 7. More formally, a test for the null hypothesis of zero correlation was carried out. No statistically significant correlation was observed. That means that the hypothesis that stated the correlation is zero cannot be rejected. Hence, there is no evidence that a pair of doctors who agreed on one type of diagnosis also agreed on the other types of diagnoses or vice versa.

## 4. Discussion 

### 4.1. Interrater Reliability of Pulse Examination

The results showed low levels of interrater reliability. A blinded study on the intra-rater reliability of pulse examination in Ayurveda reported a favorable result (*P* value = 0.02) [11]. Another blinded controlled study also reported low levels of intra- and interrater reliability with moderate kappa values for the group of experienced doctors [21]. The hypothesis of random rating was rejected for the overall test using the average pairwise kappa. According to this, the interrater agreement can be considered better than random rating. However, the practical relevance of this can be disputed in light of the small average kappa value of only 0.07 since just one pair-wise kappa was statistically significant.

Similarly, in traditional Chinese medicine and traditional Japanese Toyohari medicine, studies on pulse examination showed results ranging from a low to a good level of reliability [22]. In most of the studies, the identified reasons behind the low level of reliability were difficult pulse terminology and lack of a standard pulse-taking procedure. Furthermore, efforts are being made to improve the reliability of traditional Chinese medicine (TCM) practitioners by standardizing pulse examination procedures [23]. In Ayurveda, the low level of reliability could be due to lack of a standardized pulse-taking procedure, proper training, and experience. Other possible factors that influence the reliability of pulse examination are school of thought and understanding of the construct. In Ayurveda, pulse diagnosis has two major schools: one focuses on the “position of fingers” to assess *dosha* dominance at respective fingers, while another school assesses nature and type of flow and status (temperature, texture, and feel) of artery irrespective of finger positions. 

### 4.2. Interrater Reliability of Tongue Diagnosis

The overall reliability for tongue diagnosis ranged from poor to moderate levels. Similarly, in TCM, interrater reliability was low (no formal statistical analysis used) for tongue examination [22]. In another TCM study, three practitioners examined subjects' tongues in forty-five otherwise healthy subjects with hypercholesterolemia. Levels of interrater reliability were low (kappa = 0.22) for tongue coating reliability of three of the practitioners, whereas the level of reliability was high (kappa = 0.87) for at least two of the practitioners [24]. In Ayurveda, the low level of reliability for tongue examination could be due to a lack of a standardized tongue examination procedure. The cause of the low reliability may be a lack of specific terminology to differentiate between a thin and a thick coating. In TCM, an evidence-based standard was developed to evaluate the thin and thick tongue coating [25]. In Ayurveda, future studies and clinical training should utilize precise diagnostic procedures to improve reliability of tongue diagnosis. 

As for tongue diagnosis, despite the rather small value 0.17 of the average kappa, the hypothesis of random rating was rejected for the overall test using the average pairwise kappa.

### 4.3. Interrater Reliability of *Prakriti* Assessment

In comparison with the pulse and tongue diagnosis, the reliability of the *prakriti* assessment showed a poor to substantial level of reliability. The hypothesis of random rating was rejected for 12 pairwise kappas and also based on the average kappa value which was 0.28. Nevertheless, given that the *prakriti* assessment involved all diagnostic methods, observation, touch, and questioning, more favorable results could be expected. It is necessary to identify the cause behind this low interrater variability. Various factors could affect the consistency of *prakriti* assessment. For instance, all *prakriti* parameters are grouped into physical, physiological, and psychological factors (Table 1). The number of parameters considered for *prakriti* assessment may vary from doctor to doctor, which increased the assessment variability. Furthermore, the possibility of skipping important parameters and/or questions might lead to a different assessment. A difference in the quantification of physical parameters such as BMI or facial metrics is a possible explanation for diagnosis variance. For instance, in Sasang medicine, researchers have been attempting to develop objective and reasonable methods of determining constitutions [26]. Similarly, in Ayurveda, the combination of body shape, face pictures and matrices, voice recording, and a questionnaire might decrease the subjectivity of a physical assessment. On the other hand, for physiological (e.g., appetite, bowel habit) and psychological parameters (e.g., memory, anger), the doctors have to rely on the subjects' responses. Variation in the phrasing of the doctor's questions and the subject's answers may also negatively affect the consistency of diagnostic reliability. The doctor can retrieve precise answers from the subject by asking specific and more relevant questions. Furthermore, some doctors may give more importance to physical parameters than to physiological ones and some may depend on other parameters. The *prakriti* assessment is not a mechanical process designed to achieve an answer to a question; rather, the doctor has to understand and diagnose correctly by skillful observation, touch, and precise questioning. 

The present study was conducted without additional training of the doctors. It is necessary to assess the reliability of *prakriti* assessment after proper training. A study on the reliability of sasang constitutional body trunk measurement (SCBTM) strongly recommended giving comprehensive training prior to carrying out SCBTM [27].

In the present study, a comparison between the self-reported questionnaire and software and the assessment favored by most doctors was significant. The diagnosis given by the doctor was on average consistent with the questionnaire and software assessment. Hence, this suggests that there was much more variability in assessment among the doctors in comparison to the questionnaire or software. In the clinical practice, a good approach to improve the reliability of *prakriti* assessment might be to ask the patients to fill in the questionnaire or participate in the software analysis before the doctor's assessment. Later, the doctor can use his/her clinical experience to draw conclusions on the final diagnosis in the final assessment. It may be difficult for the doctors to use interviewer-assisted or interview-administered questionnaire in their busy schedules. Thus, it may be more convenient to use self-reported questionnaires in both clinical and research settings if the respondents have sufficient ability to fill in the questionnaire. The best example of a self-administered questionnaire is the WHO quality of life self-assessment questionnaire (WHOQOL-BREF). However, initial efforts should be made to standardize *prakriti* questionnaire for research purposes.

### 4.4. The Frequency Classes for All Assessments

**Figure 6:**
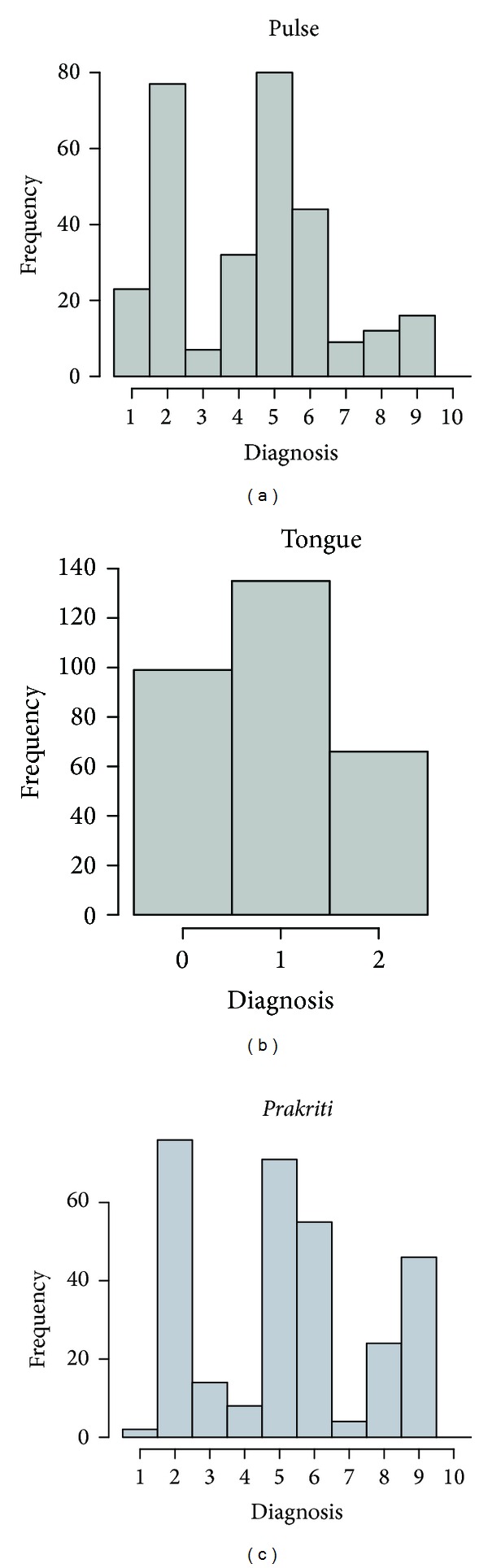
The frequencies accumulated for all doctors for pulse (a), tongue (b), and *prakriti* (c) assessment.

**Figure 7:**
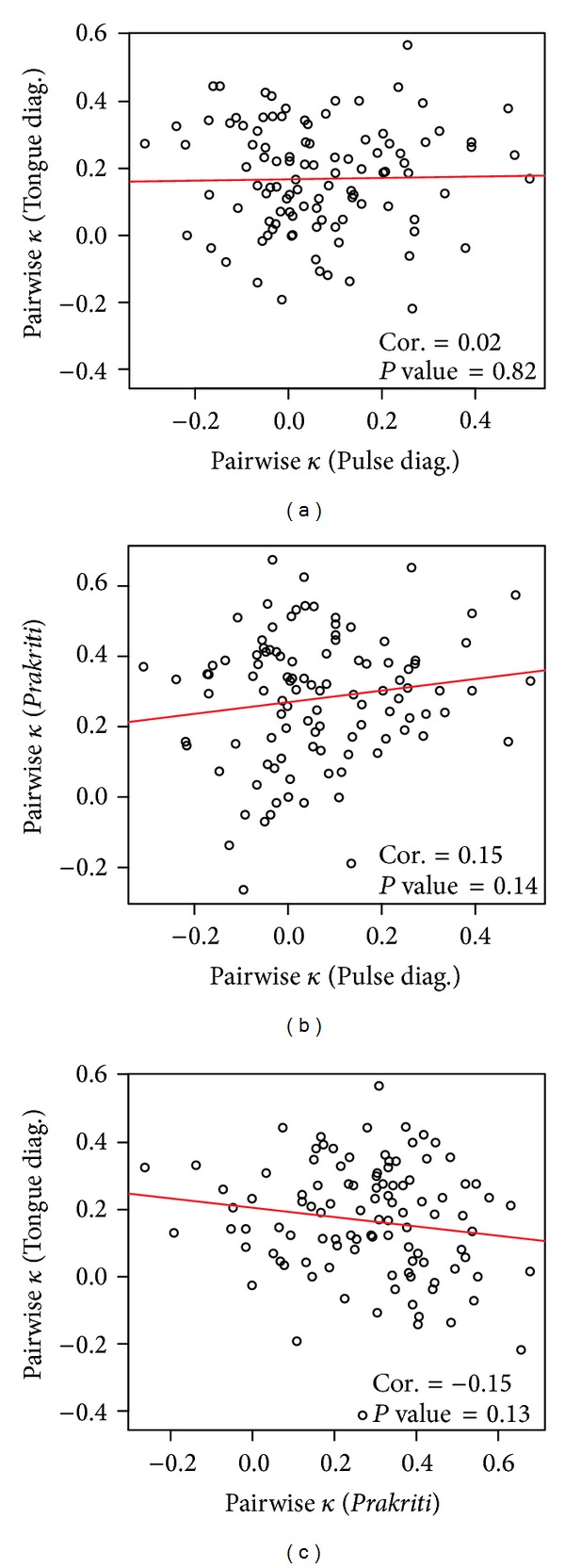
Scatter plots of the pairwise kappa values between different diagnoses. Shown in each panel are the Pearson correlation coefficient and a corresponding *P* value for the hypothesis of zero correlation coefficient.

For pulse examination, the group of *kapha* was less frequently diagnosed than the *pitta* and *vata* groups (Figure 6(a)). The reason for this may be that it is easier to sense the pulse under the first and the middle fingers than under the ring finger. Additionally, a jumping or high amplitude pulse is easier to feel than a slow, smooth movement. 

Seven different types of *prakriti* (V, P, K, VP, VK, PK, and VPK) are described in Ayurveda, but doctors also diagnosed other classes such as *pittavata, kaphapitta, *and* kaphavata* (Figure 6(c)). The term *“dwandvaja prakriti”* represents “equal” contribution of two *doshas*, while the types (e.g., PV and VP) practically represent relative dominance of *dosha*. Hence, the seven types by authentic text (*Samhitas*) become ten practical classifications of *prakriti*.

### 4.5. Factors That Influence Reliability

Various factors can affect the consistency of the diagnoses such as variability in the experience, specialization, and the schooling of the doctors. The doctors in this study had different levels of clinical experience and different specializations. Participating doctors also pointed out that an inherent variability is due to different traditional backgrounds and a lack of standardization of diagnostic methods. Another factor that influences the reliability is changeable signs and symptoms within some time frame. *Prakriti* remains unchangeable over time, while tongue coating may change, and high variability may occur in the pulse. 

### 4.6. Study Limitations

Intrarater reliability of pulse, tongue, and *prakriti* assessment was not assessed as a part of this study. Assessment of intra-rater reliability is difficult for some direct observable signs and symptoms of tongue and *prakriti* assessment, since results may be influenced by the observer's memory or attempts at consistency in observations. 

In [21], we conducted a blinded, randomized study to assess the intra-rater and interrater reliability of pulse examination as a first part of this study. Pulse characteristics may change within hours. Thus, intra-rater reliability of pulse examination should be conducted in a short time to avoid possible variation in pulse. Therefore, blinding and randomization is necessary to avoid carryover effect of the previous diagnosis. 

The number of subjects was limited to twenty to reduce chance of fatigue among the doctors. Another limitation of the study was the use of self-reported *prakriti* questionnaire. In particular, subjects may exaggerate symptoms, or they may underreport the severity or frequency of symptoms in order to generate a specific type of *prakriti*. 

## 5. Conclusions 

This is the first study to comprehensively investigate the interrater reliability of the pulse, tongue, and *prakriti* assessment used in Ayurveda. According to the LK scale and considering the separate pairwise kappas, poor to moderate levels of interrater reliability were obtained for pulse and tongue assessment. Poor to substantial levels of reliability were obtained for *prakriti *assessment. These findings are like those associated with other assessments of reliability conducted on other traditional medicine methodologies such as Chinese and Sasang medicine, where reliability has also been found to be low. We emphasize the use of an objectively defined questionnaire and software analysis in establishing a *prakriti *assessment, a method which yields more reliable results. With respect to clinical research into Ayurveda, if the body constitution assessment is to be included as an inclusion or exclusion criterion, it is necessary to establish its reliability. For all three diagnostic methods, the hypothesis of random rating was rejected based on the average kappa values. On the other hand, the average kappa values were all rather small, and so one might question whether this statistical significance is relevant from a practical point of view. For example, for pulse diagnosis, the average kappa was just 0.07 which corresponds to a very poor level of reliability.

The main reason behind the poor reliability of Ayurveda diagnosis could be lack of a systematic objective methodology and a precise operational definition of the diagnostic methods. Additional research is needed to help improve the reliability for these diagnostic methods. Furthermore, future studies on reliability should be performed after establishing objective methodology and ensuring proper training. 

In general, the interrater reliability was unimpressive, and there is room for improvement for all diagnostic methods. The best reliability of body constitution assessment was obtained when questionnaires and software were used. Accordingly, we suggest that standardization of diagnostic methods may improve the level of reliability.

## Supplementary Material

Self-assessment prakriti questionnaire. A detailed questionnaire for Prakriti assessment was developed on the basis of original Ayurveda text in simple, everyday language.

## Figures and Tables

**Table 1:** Body constitution assessment.

Diagnostic technique	Variable	Characteristic investigated	Specific characteristics for body constitution		
			*Vata *	*Pitta *	*Kapha *
Inspection	Physique	Head, forehead, face, eyes, lips, jaws, shoulder, chest, hands, palms, nails, legs, soles, joints, blood vessels, tendons.	Thin and slim physique, prominent bones	Moderately developed physique	Stout, well developed, large forehead
	Eyes	Colour and appearance	Small, dry, dull, muddy sclera, brown eyes	Reddish tinges to sclera, medium size, piercing eyes	White sclera, attractive, large eyes with thick eyelashes
	Skin	Texture, appearance, colour	Thin, hard, rough, cracked, dark, skin, superficial veins and tendons	Reddish, pink skin often with moles and acne	Thick, white, pale, yellow, oily
	Scalp hair, body hair	Texture, nature, growth, colour, body hair (colour)	Scanty, thin, coarse, dry, wavy hair	Fine, soft, red or gray hair often balding in early age	Dark, thick, dense, oily, lustrous hair
	Teeth	Size, appearance, shape and colour	Brittle/cracked, too large, uneven, dull blackish irregular	Yellowish, loose, medium	Milky white, lustrous, even large, regular
	Walking	Style, speed	Quick, fast or variable excessive unsteady	Medium, sharp accurate	Slow, firm/steady, less
	Complexion	Colour, nature	Dark complexion, Tans easily, rough, wrinkled, brittle	Reddish, pink, smooth, soft skin, sunburns easily	White, pale, tans evenly
	Voice	Quality	Low, weak, hoarse voice	High pitch, sharp voice	Deep tone, pleasant
	Speech	Content of speech	Quick, adaptable, indecisive mental nature, excessive, irrelevant in between	Intelligent, sharp, accurate, penetrative, critical, argumentative	Slow, steady, convincing, sweet and pleasing to ears, well thought of

Touch	Skin	Nature, texture, temperature	Thin, dry, cold	Warm, moist	Oily, smooth, and soft skin
	Hair	Nature, texture	Dry, hard, coarse	Soft	Smooth, oily
	Pulse	Location, pattern	Felt under the index finger like a snake crawling	Felt under the middle finger like frog's jumping	Felt under the ring finger slow, steady like pigeons movement
	Joint	Movement	Cracking sound		

Questioning	Appetite	Frequency, amount	Appetite is variable, infrequent	Strong appetite. Irritable if you miss a meal	Regular, low, can skip meals easily
	Thirst	Frequency, amount	Variable, infrequent	Strong, large amount of water needed	Low, less amount of water
	Food habits	Type of food liking, suit	Hot, oily	Cold, sweet	Hot, dry
	Bowel and bladder habit	Frequency, amount	Irregular, variable, tendency towards constipation, hard stool	Regular, tendency towards loose motion, loose soft, semisolid stool	Regular, no constipation or loose motion medium
	Strength	Physical, mental, resistance power, healing power	Low	Medium	Good
	Perspiration	Amount, odour	Scanty sweat often without smell	Profuse, hot sweat often with strong smell	Moderate, cold sweat
	Sleep	Amount, nature, quality	Light, disturbed sleep. Tends towards insomnia	Moderate sleep. Wakes up at the smallest sound sleep	Sound sleep, difficulty in waking up
	Dreams	Type of dreams	Flying, moving, restless in dreams and has nightmares	Colourful (specially red), passionate, fighting, fire in dreams	Few, sentimental water, rivers in dreams
	Body weight	Body weight changes	Low, difficulty in putting on weight	Stable	Gain easily and lose with difficulty
	Weather	Preference and tolerance	Prefer warm climate, sunshine, and moisture	Prefer cool and well ventilated place	Any climate is fine as long as it is not humid
	Working/Activity	Style, speed	Fast, quick, variable, wavering, and easily deviated	Moderate, sharp, accurate	Slow, steady, well thought of
	Psychological characteristics	Memory	Poor memory, observation good but forgets easily	Sharp, clear memory	Slow to take notice but will not forget easily
		Emotions	Anxiety, fear, uncertainty	Anger, ambitious, practical	Calm, peaceful
		Anger	Quick and unstable	Quick and sustained	Always cool
	Others	Friends	Few	Medium	More

**Table 2:** Diagnosis classes and weights for each variable.

Diagnosis classes	Types of pulse	Body constitution (*prakriti*)	Weights for each variable
*C*_1_	*Vata *	*Vataja *	(1, 0, 0)
*C*_2_	*Vatapitta *	*Vatapittaja *	(2/3, 1/3, 0)
*C*_3_	*Vatakapha *	*Vatakaphaja *	(2/3, 0, 1/3)
*C*_4_	*Pitta *	*Pittaja *	(0, 1, 0)
*C*_5_	*Pittavata *	*Pittavataja *	(1/3, 2/3, 0)
*C*_6_	*Pittakapha *	*Pittakaphaja *	(0, 2/3, 1/3)
*C*_7_	*Kapha *	*Kaphaja *	(0, 0, 1)
*C*_8_	*Kaphavata *	*Kaphavataja *	(1/3, 0, 2/3)
*C*_9_	*Kaphapitta *	*Kaphapittaja *	(0, 1/3, 2/3)
*C*_10_	*Vatapitta kapha *	*Vatapitta Kaphaja (tridoshaja) *	(1/3, 1/3, 1/3)

**Table 3:** Distance matrix between categories in *prakriti* and pulse examination.

Classes	*C*_1_	*C*_2_	*C*_3_	*C*_4_	*C*_5_	*C*_6_	*C*_7_	*C*_8_	*C*_9_	*C*_10_
*C*_1_	0	0.106	0.106	1	0.553	1	1	0.553	1	0.423
*C*_2_	0.106	0	0.2	0.55	0.2	0.6	1	0.6	0.8	0.225
*C*_3_	0.106	0.2	0	1	0.6	0.8	0.553	0.2	0.6	0.225
*C*_4_	1	0.553	1	0	0.106	0.106	1	1	0.553	0.423
*C*_5_	0.553	0.2	0.6	0.106	0	0.2	1	0.8	0.6	0.225
*C*_6_	1	0.6	0.8	0.106	0.2	0	0.553	0.6	0.2	0.225
*C*_7_	1	1	0.553	1	1	0.553	0	0.106	0.106	0.423
*C*_8_	0.553	0.6	0.2	1	0.8	0.6	0.106	0	0.2	0.225
*C*_9_	1	0.8	0.6	0.553	0.6	0.2	0.106	0.2	0	0.225
*C*_10_	0.423	0.225	0.225	0.423	0.225	0.225	0.423	0.225	0.225	0

*C*: diagnosis classes.

**Table 4:** Distances between tongue diagnoses.

Tongue coating and classes	*C*_1_	*C*_2_	*C*_3_
No coating: *C*_1_	0	0.5	1
Medium coating: *C*_2_	0.5	0	0.5
Tongue coating: *C*_3_	1	0.5	0

**Table 5:** Percentage of pairwise kappas within each LK category of reliability for pulse, tongue, and *prakriti* assessment and number of significant pairwise kappas.

Landis and Koch scale	Kappa range	Body constitution	Tongue	Pulse
Poor	(−1.0, 0.0)	9	16	40
Slight	(0.01, 0.20)	22	37	37
Fair	(0.21, 0.40)	44	41	20
Moderate	(0.41, 0.60)	22	6	3
Substantial	(0.61, 0.80)	3	0	0
Almost perfect/perfect	(0.81, 1.00)	0	0	0
Number of significant	—	12	0	1

**Table 6:** The average pairwise kappa, the corresponding *P* value, and Landis and Koch scale.

Diagnosis	Mean kappa	*P* value	LK scale
*Prakriti *	0.28	2*e* − 05	Fair
Tongue	0.17	2*e* − 05	Slight
Pulse	0.066	2*e* − 05	Slight

**Table 7:** The pairwise kappa, the *P* values, and Landis and Koch scale between the modal^#^ assessment of all doctors, software assessment, and questionnaire assessment.

	Kappa	*P* value	LK scale
Mode assessment versus Software	0.487	2*e* − 04*	Moderate
Mode assessment versus Questionnaire	0.497	0.0026*	Moderate
Software versus Questionnaire	0.336	0.0114*	Fair

^#^Modal assessment: the most frequent assessment given to the subject by doctors.

## Appendices

### A. Interpretation of Sanskrit Words

- *Dosha*: fundamental energies or entities or principles, which govern the function of body on the physical and psychological levels. The Ayurvedic concepts of physiology, pathology, diagnosis, medicine, and therapeutics are based on the doctrine of *tridoshas*.
- *Vata*: combination of air and ether elements representative of kinetic energy and movement, physical or mental functions, and degeneration.
- *Pitta*: combination of fire and water elements representing thermal energy and metabolism conversion, vision, and emotions.
- *Kapha*: combination of earth and water elements representing potential energy and structure in the body. It is associated with processes of generation, reunion, and synthesis.

### B. Body Constitution Analysis Questionnaire

For more details see supplementary material available online at http://dx.doi.org/10.1155/2013/658275.
