# Supplementary material for: Interrater Reliability of Diagnostic Methods in Traditional Indian Ayurvedic Medicine
Source: Evid Based Complement Alternat Med. 2013 Sep 26;2013:658275. doi: 10.1155/2013/658275 (PMC3803118; doi:10.1155/2013/658275)
Supplement: Supplementary file 1 — Self-assessment prakriti questionnaire. A detailed questionnaire for Prakriti assessment was developed on the basis of original Ayurveda text in simple, everyday language. [file 658275.f1.docx]

1. **Body Constitution Analysis Questionnaire**

Name –

Birthdate -

Address –

E-mail -

Occupation –

Weight –

Height –

Sex – F/M

Tick the appropriate column

| ***Vata* Test** | | | | |
| --- | --- | --- | --- | --- |
| 1. | I am lively and enthusiastic | not so much | normally medium | yes very much |
| 2. | I am talkative and fast speaking | not so much | normally medium | yes very much |
| 3. | I am easily excited | not so much | normally medium | yes very much |
| 4. | I am easily afraid or worried | not so much | normally medium | yes very much |
| 5. | I am mentally restless | not so much | normally medium | yes very much |
| 6. | My nature is wavering, changeable | not so much | normally medium | yes very much |
| 7. | I have difficulties in making decisions | not so much | normally medium | yes very much |
| 8. | I am physically very active | not so much | normally medium | yes very much |
| 9. | I walk quickly | not so much | normally medium | yes very much |
| 10. | I can grasp new things quickly | not so much | normally medium | yes very much |
| 11. | I have difficulties in memorizing | not so much | normally medium | yes very much |
| 12. | My body is rather thin, I do not put on weight easily | not so much | normally medium | yes very much |
| 13. | My weight is less, My lifelong tendency has been to be thin | not so much | normally medium | yes very much |
| 14. | My skin is dry, cool and rough, especially in winter | not so much | normally medium | yes very much |
| 15. | I get cold hands and feet often | not so much | normally medium | yes very much |
| 16. | I do not like cold weather | not so much | normally medium | yes very much |
| 17. | My hair is dry, curly | not so much | normally medium | yes very much |
| 18. | My teeth are crooked and protruded | not so much | normally medium | yes very much |
| 19. | My eyes are small and active | not so much | normally medium | yes very much |
| 20. | My lips are cracking, thin, dry | not so much | normally medium | yes very much |
| 21. | My appetite and thirst are variable | not so much | normally medium | yes very much |
| 22. | I have a tendency for hard stool and gas frequently and constipation | not so much | normally medium | yes very much |
| 23. | I do not fall asleep easily | not so much | normally medium | yes very much |
| 24. | I often wake up in the night | not so much | normally medium | yes very much |
| 25. | My dreams are often about flying, jumping, running or fearful | not so much | normally medium | yes very much |
| ***Pitta* Test** | | | | |
| 1. | I have a sharp intellect | not so much | normally medium | yes very much |
| 2. | I have a tendency to perfectionism | not so much | normally medium | yes very much |
| 3. | I like to work precisely and methodically | not so much | normally medium | yes very much |
| 4. | I like to follow my own ideas, can also be stubborn | not so much | normally medium | yes very much |
| 5. | I get impatient easily | not so much | normally medium | yes very much |
| 6. | I get easily angry | not so much | normally medium | yes very much |
| 7. | I can flare up quickly, but also calm down quickly | not so much | normally medium | yes very much |
| 8. | I am mentally quick, aggressive, intelligent | not so much | normally medium | yes very much |
| 9. | My nature is determined, fanatic sometimes | not so much | normally medium | yes very much |
| 10. | My memory is sharp | not so much | normally medium | yes very much |
| 11. | My body and weight are moderate | not so much | normally medium | yes very much |
| 12. | My activity is moderate | not so much | normally medium | yes very much |
| 13. | My skin is oily, warm, soft I have fair skin which easily sunburns, with a lot of moles | not so much | normally medium | yes very much |
| 14. | My hair has at least one of the following qualities: thin, silky, blond reddish, early gray or hair loss | not so much | normally medium | yes very much |
| 15. | My teeth are moderate in size | not so much | normally medium | yes very much |
| 16. | My eyes are sharp or penetrating | not so much | normally medium | yes very much |
| 17. | My appetite and thirst strong, excessive | not so much | normally medium | yes very much |
| 18. | My digestion is good, whatever I eat | not so much | normally medium | yes very much |
| 19. | I feel unwell, if I have to skip a meal | not so much | normally medium | yes very much |
| 20. | My eliminations are soft, oily, loose | not so much | normally medium | yes very much |
| 21. | I do not like hot weather, rather too cold than hot | not so much | normally medium | yes very much |
| 22. | I sweat easily, with strong odor | not so much | normally medium | yes very much |
| 23. | I love to have cold drinks /food | not so much | normally medium | yes very much |
| 24. | My sleep is little, but sound | not so much | normally medium | yes very much |
| 25. | My dreams are often fiery, angry, passionate, about fighting or violence or colourful | not so much | normally medium | yes very much |
| ***Kapha* Test** | | | | |
| 1. | Others find my nature agreeable | not so much | normally medium | yes very much |
| 2. | I am affectionate and tolerant | not so much | normally medium | yes very much |
| 3. | I do not get angry or irritated easily | not so much | normally medium | yes very much |
| 4. | By nature I am steady and loyal | not so much | normally medium | yes very much |
| 5. | Mentally I am calm, slow and receptive | not so much | normally medium | yes very much |
| 6. | I am peaceful by nature and not easily perturbed | not so much | normally medium | yes very much |
| 7. | I have an excellent past memory | not so much | normally medium | yes very much |
| 8. | I like to work leisurely, not in a hurry | not so much | normally medium | yes very much |
| 9. | My way of walking is slow and steady | not so much | normally medium | yes very much |
| 10. | Physically I am lethargic | not so much | normally medium | yes very much |
| 11. | My body is fairly large and athletic | not so much | normally medium | yes very much |
| 12 | My body has a tendency to put on weight easily | not so much | normally medium | yes very much |
| 13 | My skin is oily, thick, cool | not so much | normally medium | yes very much |
| 14 | My hair is oily, thick, wavy | not so much | normally medium | yes very much |
| 15 | My teeth are strong, white and well-formed | not so much | normally medium | yes very much |
| 16 | My are big, attractive or with thick eye lashes | not so much | normally medium | yes very much |
| 17 | My appetite is slow, but steady | not so much | normally medium | yes very much |
| 18 | When I am busy, I can easily skip a meal | not so much | normally medium | yes very much |
| 19 | My eliminations are thick, oily, heavy, slow | not so much | normally medium | yes very much |
| 20 | I have a tendency of fullness in stomach | not so much | normally medium | yes very much |
| 21 | I don’t like cold and rainy weather | not so much | normally medium | yes very much |
| 22 | I prefer warm food and drinks | not so much | normally medium | yes very much |
| 23 | My sleep is heavy, prolonged, excessive | not so much | normally medium | yes very much |
| 24 | With less than 8 hours of sleep I don’t feel fit during the day | not so much | normally medium | yes very much |
| 25 | My dreams are often about water, ocean, swimming or romantic | not so much | normally medium | yes very much |

This questionnaire is based on

1. Y. T. Acharya, “*Sushruta Samhita*,” Chaukhamba Surbharati, Varanasi, India, Sharira Sthan 4, 64-77, 2003.

Y. T. Acharya, “*Caraka Samhita*,” Chaukhamba Surbharati, Varanasi, India, Viman Sthan 8/96-98, 1992.
